# A Real-World, Multicenter Assessment of Drugs Requiring Weight-Based Calculations in Overweight, Adult Critically Ill Patients

**DOI:** 10.1155/2013/909135

**Published:** 2013-11-30

**Authors:** Sandra L. Kane-Gill, Nicholas P. Wytiaz, Lisa M. Thompson, Karina Muzykovsky, Mitchell S. Buckley, Henry Cohen, Amy L. Seybert

**Affiliations:** ^1^Department of Pharmacy, Translational Science Institute and Critical Care Medicine, Schools for Pharmacy and Medicine, University of Pittsburgh, 3501 Terrace Street, Pittsburgh, PA 15261, USA; ^2^Department of Pharmacy, University of Pittsburgh Medical Center, Pittsburgh, PA 15213, USA; ^3^West Penn Allegheny Health System, The Western Pennsylvania Hospital, 4800 Friendship Avenue, Pittsburgh, PA 15224, USA; ^4^Banner MD Anderson Cancer Center, 2946 E. Banner Gateway Drive, Gilbert, AZ 85234, USA; ^5^The Brooklyn Hospital Center, 121 DeKalb Avenue, Brooklyn, NY 11201, USA; ^6^Banner Good Samaritan Medical Center, Department of Pharmacy, 1111 E. McDowell Road, Phoenix, AZ 85006, USA; ^7^Arnold & Marie Schwartz College of Pharmacy and Health Sciences, Long Island University, 75 DeKalb Avenue, Brooklyn, NY 11201, USA; ^8^Kingsbrook Jewish Medical Center, 585 Schenectady Avenue, Brooklyn, NY 11203, USA; ^9^University of Pittsburgh, School of Pharmacy, 3501 Terrace Street, Pittsburgh, PA 15261, USA

## Abstract

Prescribing appropriate doses of drugs requiring weight-based dosing is challenging in overweight patients due to a lack of data. With 68% of the US population considered overweight and these patients being at an increased risk for hospitalization, clinicians need guidance on dosing weight-based drugs. The purpose of this study was to identify “real-world” dose ranges of high-risk medications administered via continuous infusion requiring weight-based dosing and determine the reasons for dosing changes (ineffectiveness or adverse drug reactions). A prospective, multicenter, observational study was conducted in four intensive care units at three institutions. A total of 857 medication orders representing 11 different high-risk medications in 173 patients were reviewed. It was noted that dosing did not increase in proportion to weight classification. Overall, 14 adverse drug reactions occurred in nine patients with more in overweight patients (9 of 14). A total of 75% of orders were discontinued due to ineffectiveness in groups with higher body mass indexes. Ineffectiveness leads to dosing adjustments resulting in the opportunity for medication errors. Also, the frequent dosing changes further demonstrate our lack of knowledge of appropriate dosing for this population. Given the medications' increased propensity to cause harm, institutions should aggressively monitor these medications in overweight patients.

## 1. Introduction

The 2008 National Health and Nutrition Examination Survey indicates that 68% of the US adult population is overweight [1]. Overweight men and women have a higher number of hospital admissions compared to normal weight persons [2, 3]. With the increased number of admissions, clinicians are encountering new management challenges when providing care for these patients. Prescribing appropriate doses of medications such as opioids, anticoagulants, thrombolytics, anti-infectives, cardiac agents, corticosteroids, anticonvulsants, neuromuscular blocking agents, and sedatives in this overweight population is challenging since weight-based dosing is necessary and limited data addressing optimal dosages are available [4–8].

Clinicians, in particular pharmacists, rely on interpreting the pharmacokinetic properties of drugs requiring weight-based dosing to estimate the correct dosages when specific dosage recommendations are lacking [4–9]. Inappropriate dosing is a concern due to the possibility of therapeutic failure from underdosing and adverse drug reactions (ADRs) associated with overdosing. Interestingly, many of the drugs requiring weight-based dosing are the same drugs on the List of High-Alert Medications published by the Institute for Safe Medication Practices (ISMP) [10]. High-alert medications are drugs with a heightened risk of causing significant patient harm when used in error. Inappropriate dosages of weight-based drugs are considered a medication error that could contribute to patient harm.

The purpose of this evaluation was to identify “real-world” dose ranges of high-risk medications administered via continuous infusion requiring weight-based dosing used in overweight populations and establish a foundation for standardized, institution-specific dosing guidelines for these patients.

## 2. Methods

This was a prospective, multicenter, observational study. Participating sites were the Cardiac Intensive Care Unit (CICU) and Medical Intensive Care Unit (MICU) of the University of Pittsburgh Medical Center (UPMC) Presbyterian Hospital (Pittsburgh, PA); the MICU of Kingsbrook Jewish Medical Center (Brooklyn, NY); and the CICU of Banner Good Samaritan Medical Center (Phoenix, AZ). UPMC Presbyterian is an adult tertiary academic medical center with over 800 licensed inpatient beds, including a 10-bed CICU and 24-bed MICU. Banner Good Samaritan is a quaternary care, teaching hospital with over 650 licensed inpatient beds, including a 16-bed CICU. Kingsbrook Jewish Medical Center is a teaching, nonprofit, private community institution with over 300 licensed inpatient beds, including a 10-bed MICU.

Data were collected for a continuous sample of patients >18 years of age admitted to any of the designated units during a 6-week period who received one or more high-risk medication, as defined by the Institute for Safe Medication Practices List of High-Alert Medications [10]. Exclusion criteria were as follows: one time orders, medications given on a scheduled basis (daily, BID, Q6 h, Q8 h, etc.), medications not requiring weight-based dosing, missing information necessary for calculation of BMI (height, weight), medications ordered but not given, bolus doses of medications, and patients with renal dysfunction (dialysis or creatinine clearance (CrCl) < 30 mL/min) and/or liver failure (Child's Pugh grade of C).

### 2.1. Data Collection

After IRB approval at the three institutions data were collected. Every day during the 6-week period, new medication orders, change in rate orders, and discontinued orders for the target high-risk medications were evaluated. Orders on the weekends were evaluated on Monday. All information was obtained from the patient's electronic medical chart. Identifiable information was not collected to ensure compliance with Health Insurance Portability and Accountability Act (HIPPA) regulations. Patient data included sex, age, race, height, weight, dialysis use, liver panel, and serum creatinine. Drug data were obtained daily for new medication orders and changes in doses including drug name, dose, concentration, route, and rate. Discontinued orders were evaluated daily for reasons of discontinuation by reviewing clinician notes (physician and nurses) and communication with clinicians. Reasons of interest for discontinuation were ineffective dose, weaning from drug, or potential ADR and undeterminable. When a potential ADR was identified as a reason for drug discontinuation, then these potential ADRs were evaluated and classified using three published, objective causality assessment tools (modified-Kramer, Naranjo et al., and Jones) [23–25]. Any drug-related adverse event identified required at least two of the three causality instruments to suggest the likelihood of an ADR by having a score of “possible, probable or definite” to be included in our analysis. This method for ADR evaluation has been used previously [26]. Consistent with the definition used for the causality instruments, an ADR was defined as “an undesirable clinical manifestation that is consequent to and caused by the administration of a particular drug” [27].

From the collected data, additional values were calculated for each patient including CrCl via Cockcroft and Gault, Child's Pugh, and body mass index (BMI) [28]. Patients were then categorized based on their calculated BMI. The World Health Organization and National Institute for Health definitions were used when categorizing patients by BMI [29, 30]. The five categories are as follows: underweight (<18.5 kg/m^2^), normal weight (18.5–24.9 kg/m^2^), overweight (25–29.9 kg/m^2^), obese (30–39.9 kg/m^2^), and extremely obese (>40 kg/m^2^).

Data compiled from all three sites were grouped together and divided by drug. Descriptive statistics were analyzed using SPSS v. 18. (Chicago, IL). For drugs with multiple rate changes identified in the daily data collection, data were recorded for the last dose received in the previous 24-hour period. Medications with the most orders (>15) were assessed. Our analysis included vasoactive drugs (dobutamine, dopamine, milrinone, nitroglycerin, and phenylephrine), heparin, sedatives (propofol, midazolam, and fentanyl), and rocuronium. Medication dosing identified in this real-world evaluation was compared to recommendations in the package insert for each drug.

## 3. Results

A total of 857 medication orders representing 11 different high-risk medications in 173 patients were reviewed (underweight = 4, normal = 41, overweight = 60, obese = 60, and extremely obese = 31).

The dosing results for vasoactive agents are provided in Table 1 [11–16]. There were 263 doses evaluated for six different vasoactive medications in 78 patients. Across all weight categories, dose ranges greatly varied for each drug and were as follows: dobutamine 2.29–10.05 mcg/kg/min, dopamine 1–20 mcg/kg/min, milrinone 0.19–1.04 mcg/kg/min, nitroglycerin 0.06–0.85 mcg/kg/min, norepinephrine 0.007–0.33 mcg/kg/min, and phenylephrine 0.10–2 mcg/kg/min. Despite the wide ranges, doses for all the vasoactive medications were within the normal dosing range and/or under the maximum dose (as defined by package insert or clinical recommendation) for their respective drug. In addition, dosing did not necessarily increase in proportion to weight classification. The highest average doses were seen in normal (dopamine, norepinephrine), overweight (phenylephrine), obese (dobutamine), and morbidly obese (milrinone, nitroglycerin).

There were 162 doses evaluated for heparin in 43 patients. The dose range, across all weight categories, was 3.93–26 units/kg/hr. Maximum doses in each weight category met or exceeded the normal dosing range and/or recommended maximum dose as defined by package insert or clinical recommendation as shown in Table 2 [17, 18]. Of patients receiving heparin, the highest average dose (17.6 units/kg/hr) was seen in the normal weight category.

There were 209 doses evaluated for three different sedatives (fentanyl, midazolam, and propofol) and one neuromuscular blocker (rocuronium) in 53 unique patients. Across all weight categories, dose ranges again greatly varied for each drug and were as follows: fentanyl 0.0011–0.04 mcg/kg/min, midazolam 0.02–20 mcg/kg/min, propofol 5–101.67 mcg/kg/min, and rocuronium 3–12 mcg/kg/min. The maximum doses for propofol, midazolam, and rocuronium, regardless of weight category, exceeded the normal dosing range and/or the maximum dose as defined by package insert or clinical recommendation (Table 3) [19–22]. In addition, as seen with the vasoactive drugs, dosing did not necessarily increase in proportion to weight classification. The highest average doses were seen in the overweight (propofol), obese (midazolam), and extremely obese (fentanyl) categories.

Overall, 14 ADRs occurred in nine patients as shown in Table 4. Five of the high-risk medications were associated with an ADR. Adverse drug reactions were more common in overweight patients (9 of 14). However, most dosing regimens used in these instances were not considered exceeding the recommended dose. Only two ADRs, both involving heparin, were administered at doses greater than recommended. We evaluated all discontinued orders and the reasons for discontinued orders, other than the occurrence of an ADR; these included ineffective dose/medication (*n* = 324), weaning of dose/medication (*n* = 189), adjustment of dose per hospital protocol (i.e., heparin nomogram) (*n* = 70), and unknown reasons (*n* = 2). When assessed by weight category, orders discontinued due to ineffectiveness were most often in the obese population (35.4% (115/324)). Distribution of ineffective discontinued orders within the other weight classes was as follows: underweight (0.9% (3/324)), normal (23.7% (77/324)), overweight (22.8% (74/324)), and extreme obesity (17% (55/324)). So, in total 75% of orders evaluated were discontinued due to ineffectiveness in groups with a higher BMI, as compared to only 23.7% in normal weight patients, thus indicating the need for more frequent titration and plausibly higher doses than those for normal weight patients.

## 4. Discussion

The concern for inappropriate dosing of weight-based medications in overweight patients is truly a patient safety concern, leading to therapeutic failures or ADRs [31, 32]. Decreased awareness and limited information of optimal dosing strategies in overweight patients may contribute to inappropriate prescribing in these special populations [33]. In fact, clinician opinion even varies about which weight, ideal or actual, to use for dosing calculations, so clinicians make educated dosing guesstimates based on the pharmacokinetic properties of drugs [33]. Our evaluation emphasizes the wide variance in doses for drugs administered via continuous infusion used among different weight classes in a real-world critical care setting.

Fourteen ADRs in 9 patients related to five different medications were identified. There was a tendency for the ADRs to occur in overweight patients (12/14), but this does not necessarily appear to be a result of higher doses used in this population. It could be explained by more overweight patients included in this evaluation. Several other factors, such as severity of illness and concomitant drug therapy, could have contributed in part to these ADRs [34]. The sparse availability of documented patient weights in previous, retrospective medical record review studies has precluded a thorough assessment of weight as a risk factor for ADRs [34]. This study does provide us with the inclination that the drugs we investigated in this study are high risk and susceptible to ADRs, irrespective of weight-based dosing selections.

Weight-based dosing strategies for vasoactive medications have been suggested based on the drugs' pharmacokinetics. Since all inotropes and vasopressors, with the exception of milrinone, have short half-lives, fast onsets, and low volumes of distribution, the use of ideal body-weight (IBW) has been suggested for all weight-based vasoactive drugs [5]. Due to the frequent and rapid titration of these agents to a predetermined clinical effect, the lower starting dose provided by an ideal body weight-based dose seems to be a safer and reasonable strategy. While the package insert for vasoactive drugs has recommended weight-based dosing guidelines, these recommendations are not always abided by in real-world clinical practice and vary among institutions. The appropriate weight (actual, adjusted, or ideal) for the optimal dosing strategy in special populations (e.g., obese patients) remains unknown. Of note, the vasoactive drugs were in the dosing ranges provided by the package inserts regardless of weight classification [11–16]. The three ADRs seen in our study with the vasoactives (two with dobutamine, one with norepinephrine) occurred in a morbidly obese, an underweight, and an overweight individual. In each case, the doses identified were below the respective recommended maximum dose. Unfortunately, a recommendation for optimal weight-based vasoactive dosing strategies remains elusive in overweight populations.

Heparin was another “high-risk” medication associated with variable and inappropriate dosing strategies. In our study, heparin was dosed outside the recommendations in the package insert for all weight categories except in “underweight” patients [17]. Heparin, an anticoagulant with a volume of distribution approximating blood volume (40–70 mL/kg), is not fully distributed into adipose tissue. Optimal dosing in obesity continues to challenge clinicians. Although these patients tend to have a greater total body mass, this may not always translate into increased lean body mass compared to normal weight individuals. Dosing based on IBW risks subtherapeutic concentrations while using actual body weight (ABW) risks supratherapeutic concentrations [35, 36]. It is important to emphasize that heparin dosing strategies also vary with the therapeutic indication. Additionally, many institutions have adopted heparin dosing protocols for each indication based on the various published nomograms for the treatment of venous thromboembolism (VTE), acute coronary syndrome (ACS), and stroke. When following the heparin nomogram for obese patients, a delay in time to achieve an adequate pharmacodynamic effect has been reported [37, 38]. It has been noted that prescribers have a tendency to deviate from nomograms for obese patients [38, 39].

It is important to emphasize that the American College of Chest Physicians (ACCP) recommends an initial IV bolus dose of 80 units/kg or 5,000 units with an initial continuous infusion of 18 units/kg/hr or 1,300 units/hr for the treatment of VTE. For the treatment of ACS (NSTEMI, unstable angina, and STEMI), ACCP recommends an initial IV bolus dose of 60 units/kg (maximum 4,000 units) and an initial continuous IV infusion of 12 units/kg/hr (maximum 1,000 units/hr) [40]. Both current regimens utilize ABW for dosing. Still, several alternative dosing regimens have been developed based on IBW, dosing weight, modified dosing weight (average of ABW and IBW), and ABW with a maximum initial bolus dose. A review of the studies used to develop these alternate dosing strategies suggests that ABW is the preferred means of dosing heparin for nonmorbidly obese patients. The dosing limits set by ACCP for VTE and ACS are controversial in morbidly obese patients due to the risk of underdosing. However, four of the five ADRs associated with heparin seen in our study involved overweight or obese patients, suggesting overdosing. Regardless, data and comparative evidence for different types of weight-based strategies in overweight, as well as underweight, patients are limited [6]. Given the lack of evidence, such patients should be evaluated and dosed on an individual basis.

As with the vasoactives, sedative dosing guidelines are not always applicable and may be titrated to a desired clinical endpoint based on a patient's specific situation (mechanical ventilation, deep sedation). Much higher sedative doses are often seen in ICU patients compared to those in non-ICU patients [7]. In our study, sedatives were often dosed outside the recommendations in the package insert for all weight categories [19–22]. The sedatives reviewed in this study (midazolam and propofol) are both short-acting, hepatically metabolized, renally eliminated medications. Midazolam, a benzodiazepine, is converted to an active metabolite (1-hydroxymidazolam glucuronide) with central nervous system (CNS) depressant effects, which may accumulate in critically ill patients. Also, midazolam is highly lipophilic leading to a greater accumulation in obese patients and prolonged sedation [41]. The two ADRs with midazolam seen in our study involved overweight patients. Therefore, obese patients may benefit from lower initial doses of midazolam and daily sedation interruption [42].

Propofol, the other sedative reviewed in our study, is conjugated to inactive metabolites in the liver. The risk for prolonged sedation and CNS depression is less than that of midazolam, which is longer acting and has an active metabolite. Still, there is deeper anesthesia and concern for delayed awakenings by anesthesiologists for overweight patients [43]. ADRs were not observed with propofol in the ICU population in our study.

Fentanyl, a synthetic narcotic analgesic, is the preferred agent for agitated critically ill patients [44]. Dosing based on IBW has been suggested for all patients given the similar pharmacokinetics in obese and nonobese individuals [45]. Since measured total body clearance of the drug has a nonlinear relationship to total body weight (TBW), dosing based on TBW may result in supratherapeutic doses in obese patients.

Rocuronium, a neuromuscular blocking agent, is commonly dosed according to IBW in both obese and nonobese patients [46]. Dosing according to TBW carries the risk of prolonged duration of action in obese patients due to increased distribution and protein binding and decreased clearance compared to leaner patients. However, due to the low lipophilicity of rocuronium, its pharmacokinetic parameters are relatively similar between obese and lean patients when dosed based on IBW [46]. The four ADRs seen with rocuronium in our study occurred in one overweight individual. However, the doses identified were well below the recommended maximum.

The goal of our evaluation was to provide specific dosing guidance and precautions about ADRs and therapeutic failures for an overweight population based on real-world application. Despite a multicenter approach to achieve an adequate sample, the wide variety in patients' weights and variations in dosing preclude us from providing specific dosing recommendations. However, we did notice that patients with higher BMIs had a higher frequency of dose discontinuation due to ineffectiveness. The reason for more frequent dosing titration due to ineffectiveness in patients with higher BMIs despite the dosing being within the package insert recommendations may be due to clinicians using weight strategies such as IBW, LBM, or adjusted body weight that may not be reliable or using lower doses than TBW based on intuition to minimize the risk of toxicity. Notably, more dosing changes is an added patient safety concern with more opportunity for errors including calculation errors [47]. We do recommend that patients with higher BMIs require more vigilant monitoring for efficacy, medication errors, and ADRs. A multicenter registry for dosing of weight-based drugs in critically ill patients could be of great value for future guidance of dosing and safety precautions.

### 4.1. Limitations

This study has several limitations. First, our sample size was small as we only looked at medications for 173 unique patients receiving 10 frequently used high-risk medications over a six-week period, despite our substantial efforts including four ICUs in three institutions. We did evaluate high-risk drugs according to the ISMP's list; however we did not report the results for drugs such as diazepam, digoxin, enoxaparin, eptifibatide, or morphine. For these medications, less than 15 orders were available for analysis after the dosing exclusion criteria (scheduled regimens) were applied, thus making conclusions about dosing from such a small sample challenging. Second, because this was an observational study, it was difficult to control for confounding factors. While we did exclude certain patients from the study, such as those with renal and/or hepatic failure, we could not account for some other confounders. These factors include additional disease states, severity of illness, and concomitant medications. Third, we were unaware of the type of weight used for dosing the study patients. While we recorded the patients' actual body weight during data collection, this may not always have been the weight used for dosing by the clinician. IBW, adjusted bodyweight, and total body weight are all used in clinical practice depending on a medication's pharmacokinetic parameters. Given the various dosing weights, we attempted to standardize our data by recording the patients' actual body weight and reporting recommended dosing regimens in terms of actual body weight. Finally, doses were difficult to record for some medications such as vasoactive drugs, which are constantly being titrated to a desired clinical effect. In order to control, in part, for these frequent dose changes, data were recorded for the last dose received by a patient in a 24-hour period. This precaution limited the amount of data recorded for each patient in order to avoid skewing the average dose and range. The emphasis of this study was assessment of dosing, so we did evaluate daily doses and their impact of ineffectiveness and ADRs, thus including more than one dose per patient.

## 5. Conclusion

A wide variance was seen in the doses provided by continuous infusion of high-risk medications used across different weight classifications in critically ill adult patients. The vasoactive drugs were within the dosing range provided in the package inserts, regardless of weight classification; while heparin and the sedatives were typically dosed outside the recommendations. The number of ADRs cannot be overlooked as there was a tendency for the ADRs to occur in overweight patients, but this does not necessarily appear to be a function of higher doses used based on weight. Still, the medications reviewed in this study are commonly associated with ADRs and have been labeled as high-risk drugs by the ISMP. The frequency of dosing changes due to ineffectiveness in patients with higher BMIs presents additional safety concerns. Given the medications' increased propensity to cause harm, institutions should aggressively monitor these medications; especially in overweight patients. In order to advance the literature and provide specific dosing recommendations, we encourage the development of registries at individual institutions to track dosing and associated outcomes (ineffectiveness and ADRs) in overweight patients.

## Figures and Tables

**Table 1:** Dosing results for vasoactive drugs by weight category compared to recommendations in the literature.

Drugs	Weight category	Patients evaluated	Doses	Mean dose (mcg/kg/min)	Median Dose (mcg/kg/min)	Minimum (mcg/kg/min)	Maximum (mcg/kg/min)	Literature dose recommendation [11–16]
Dobutamine	Underweight	1	1	2.50	2.50	2.50	2.50	**Inotropic support** 2.5–15 mcg/kg/min Maximum: 40 mcg/kg/min
	Normal weight	4	7	4.11	2.76	2.29	10.05	
	Overweight	1	1	2.52	2.52	2.52	2.52	
	Obese	1	2	7.50	7.50	5.00	5.00	
	Extremely obese	3	6	3.26	3.00	2.50	5.00	

Dopamine	Underweight	1	4	6.75	7.50	4.00	8.01	**Shock/hypotension** 2–50 mcg/kg/min Titrate to desired effect (i.e., mean arterial pressure >65 mmHg)
	Normal weight	3	20	7.78	7.01	1.04	15.69	
	Overweight	9	21	4.80	5.00	1.00	10.00	
	Obese	4	12	7.45	5.00	2.49	20.00	
	Extremely obese	3	17	5.09	5.00	1.00	13.87	

	Underweight	0	0	—	—	—	—	**Inotropic support** Initial: 50 mcg/kg Maintenance: 0.375–0.75 mcg/kg/min Maximum: 1.13 mg/kg/day CrCl* < 50*: 0.43 CrCl * < 40: 0.38 *
	Normal weight	3	11	0.36	0.37	0.19	0.50	
Milrinone	Overweight	2	3	0.43	0.50	0.30	0.50	
	Obese	1	4	0.27	0.25	0.20	0.38	
	Extremely obese	2	3	0.77	0.78	0.51	1.04	

Nitroglycerin	Underweight	0	0	—	—	—	—	**Acute myocardial infarction** Initial: 0.20 mcg/kg/min Usual range: 0.20–1.50 mcg/kg/min
	Normal weight	2	4	0.28	0.24	0.17	0.47	
	Overweight	8	15	0.34	0.26	0.06	0.85	
	Obese	4	4	0.26	0.24	0.07	0.47	
	Extremely obese	1	1	0.36	0.36	0.36	0.36	

Norepinephrine	Underweight	1	5	0.19	0.22	0.06	0.33	**Shock** Initial: 0.01–3.00 mcg/kg/min Titrate to desired response (i.e., mean arterial pressure >65 mmHg) Usual Range: 8–30 mcg/min
	Normal weight	3	12	0.10	0.10	0.07	0.14	
	Overweight	4	4	0.14	0.15	0.06	0.14	
	Obese	5	30	0.06	0.04	0.01	0.30	
	Extremely obese	2	10	0.05	0.04	0.02	0.07	

Phenylephrine	Underweight	0	0	—	—	—	—	**Shock** Initial: 100–500 mcg IV bolus IV infusion: 0.50 mcg/kg/min Titrate to desired effect Reported ranges: 0.40–9.10 mcg/kg/min
	Normal weight	2	23	0.66	0.69	0.14	1.10	
	Overweight	4	6	0.84	0.59	0.45	2.00	
	Obese	0	0	NA	NA	NA	NA	
	Extremely obese	2	2	0.83	0.83	0.10	1.56	

**Table 2:** Dosing results for heparin by weight category compared to recommendations in the literature.

Drugs	Weight category	Patients evaluated	Doses	Average dose	Median dose	Minimum	Maximum	Literature dose recommendation [17, 18]
	Underweight	0	0	—	—	—	—	**VTE** 80 units/kg then 18 units/kg/hr Maximum bolus 10,000 units Maximum initial rate 1600 units/hr **ASC** 60 units/kg then 12 units/kg/hr Maximum bolus 5000 units. Max initial rate 1000 u/hr
	Normal weight	11	52	17.61	17.41	9.29	26.00	
Heparin (units/kg/hr)	Overweight	17	39	12.23	11.97	6.00	18.00	
	Obese	9	34	12.87	11.67	3.93	22.00	
	Extremely obese	6	37	11.77	10.00	4.50	21.80	

**Table 3:** Dosing results for sedatives and neuromuscular blocker by weight category compared to recommendations in the literature.

Drugs	Weight category	Patients evaluated	Doses	Average dose	Median dose	Minimum	Maximum	Literature dose recommendation [19–22] REF package insert
Propofol (mcg/kg/min)	Underweight	1	4	17.50	20.00	10.00	20.00	**ICU sedation** Initial: 5 mcg/kg/min Maintenance: 5–50 mcg/kg/min or higher to achieve a target sedation score
	Normal weight	5	14	36.67	35.50	10.00	85.50	
	Overweight	6	44	45.83	50.00	6.50	75.00	
	Obese	4	50	36.67	37.50	5.00	60.00	
	Extremely obese	7	20	21.17	10.17	8.39	101.67	

Midazolam (mcg/kg/min)	Underweight	0	0	—	—	—	—	Loading dose: 0.17–0.83 mcg/kg/min Maintenance: 0.33–1.67 mcg/kg/min
	Normal weight	3	3	0.26	0.06	0.02	0.69	
	Overweight	1	1	0.06	0.06	0.06	0.06	
	Obese	3	28	8.81	7.50	0.14	20.00	
	Extremely obese	1	6	0.24	0.26	0.06	0.41	

Fentanyl (mcg/kg/min)	Underweight	0	0	—	—	—	—	Mechanically-ventilated patients: 0.01–0.17 mcg/kg/min
	Normal weight	5	8	<0.01	<0.01	<0.01	0.01	
	Overweight	8	15	0.01	0.01	<0.01	0.01	
	Obese	5	9	0.02	0.02	0.02	0.02	
	Extremely obese	4	7	0.03	0.03	0.02	0.04	

Rocuronium (mcg/kg/min)	Underweight	0	0	—	—	—	—	**Tracheal intubation** 450–600 mcg/kg **Rapid sequence intubation** Load: 600–1200 mcg/kg IV Continuous infusion: 8–12 mcg/kg/min
	Normal weight	0	0	—	—	—	—	
	Overweight	0	0	—	—	—	—	
	Obese	1	35	7.51	7.00	3.00	12.00	
	Extremely obese	0	0	—	—	—	—	

**Table 4:** Adverse drug reactions reported.

Drug	ADR (*n* = 14 ADRs in 9 patients)	Weight category	Dosage	Weight-based dosage
Midazolam	Slightly responsive to noxious stimuli	Overweight	685.00 mcg/min	5.00 mcg/kg/min

Midazolam	Unresponsive to noxious stimuli	Overweight	2740.00 mcg/min	20.00 mcg/kg/min

Dobutamine	Ventricular tachycardia/implantable cardioverter-defibrillator firing	Underweight	386.77 mcg/min	5.00 mcg/kg/min

Dobutamine	Sinus tachycardia/implantable cardioverter-defibrillator fired	Morbidly obese	805.33 mcg/min	5.00 mcg/kg/min

Heparin	Bleeding	Normal weight	876.00 units/hr	11.20 units/kg/hr

Heparin	aPTT > 200	Overweight	3000.00 units/hr	25 units/kg/hr

Heparin	Bleeding	Obese	1150.00 units/hr	7.67 units/kg/hr

Heparin	Bleeding	Obese	1150.00 units/hr	7.67 units/kg/hr

Heparin	Bleeding	Overweight	2200.00 units/hr	18.33 units/kg/hr

Norepinephrine	Sinus bradycardia	Overweight	2.00 mcg/min	0.01 mcg/kg/min

Rocuronium	Peripheral nerve stimulation = 0	Overweight	27.33 mcg/min	0.20 mcg/kg/min

Rocuronium	Peripheral nerve stimulation = 0	Overweight	18.33 mcg/min	0.13 mcg/kg/min

Rocuronium	Peripheral nerve stimulation = 0	Overweight	16.00 mcg/min	0.12 mcg/kg/min

Rocuronium	Peripheral nerve stimulation = 0	Overweight	22.83 mcg/min	0.17 mcg/kg/min
